# Quality Control Dissolution Data Is Biopredictive for a Modified Release Ropinirole Formulation: Virtual Experiment with the Use of Re-Developed and Verified PBPK Model

**DOI:** 10.3390/pharmaceutics14071514

**Published:** 2022-07-21

**Authors:** Olha Shuklinova, Przemysław Dorożyński, Piotr Kulinowski, Sebastian Polak

**Affiliations:** 1 Faculty of Pharmacy, Jagiellonian University Medical College, Medyczna 9 Street, 30-688 Kraków, Poland; sebastian.polak@uj.edu.pl; 2 Department of Drug Technology and Pharmaceutical Biotechnology, Medical University of Warsaw, Banacha 1, 02-097 Warszawa, Poland; mfdorozy@cyf-kr.edu.pl; 3 Institute of Technology, Pedagogical University of Krakow, Podchorążych 2, 30-084 Kraków, Poland; piotr.kulinowski@up.krakow.pl; 4 Simcyp Division, Certara UK Limited, Level 2-Acero, 1 Concourse Way, Sheffield S1 2BJ, UK

**Keywords:** ropinirole, Parkinson’s disease, biopredictive dissolution, physiologically based pharmacokinetic modeling

## Abstract

Physiologically based pharmacokinetic and absorption modeling are being used by industry and regulatory bodies to address various scientifically challenging questions. While there is high confidence in the prediction of exposure for the BCS class I drugs administered as immediate-release formulations, in the case of prolonged-release formulations, special attention should be given to the input dissolution data. Our goal was to develop and verify a PBPK model for a BCS class I compound, ropinirole, and check the biopredictiveness of the dissolution data for the prolonged-release formulation administered by Parkinson’s patients. The model was built based on quality control dissolution data reported in the certificates of analysis and verified with the use of data derived from five clinical trial reports. The simulated pharmacokinetic parameters being within a two-fold range of the observed values confirmed acceptable model performance, in vivo relevance of the in vitro dissolution profiles, and indirectly indicated ropinirole stable release from the formulation in the patients’ gastro-intestinal tract. Ropinirole PBPK model will be used for exploring potential clinical scenarios while developing a new formulation.

## 1. Introduction

Parkinson’s disease is a progressive neurodegenerative disorder leading to motor and nonmotor disability [1,2]. There are a number of effective therapeutic options intended for the management of symptoms; however, none of them can stop the disease’s progression [2,3]. Therefore, antiparkinsonian therapy is chronic and aims at achieving stable drug plasma concentrations without rapid fluctuations, in what further results in a stable therapeutic response [4,5]. To increase the level of patients’ compliance and keep the plasma concentration in the blood stable, the prolonged-release formulations of antiparkinsonian drugs are often developed. One example is ropinirole, a dopaminergic drug that is used in the treatment of Parkinson’s disease (PARKD) and restless leg syndrome (RLS) [6,7]. As the drug stimulates dopamine receptors, its continuous and smooth release from a formulation has crucial importance in terms of both efficacy and safety [8]. After the immediate-release tablets were first approved by the FDA in 1998, the 24-h prolonged-release formulation was developed by GSK and approved in 2008 [9]. Considering the fact that ropinirole is a valuable therapeutic agent in Parkinson’s disease, the development of new generic formulations should be expected. The new formulations are usually selected based on the biopharmaceutical or clinical behavior anticipated in healthy adults and are subsequently used in patients without consideration of the physiological differences between populations [10]. In the case of Parkinson’s disease, patient physiological parameters that could affect drug exposure can be divided into two categories: age-related and disease-related. Regarding the latter, Parkinson’s disease is considered to be a systemic disease that changes gastro-intestinal (GI) conditions, which are crucial for drug absorption. However, these changes are often described as symptoms rather than as parameters, which makes it challenging to characterize them quantitatively [10]. Such an attempt was undertaken by Wollmer and Klein, who reviewed patient-specific GI parameters with the aim to create a platform for in vitro models for predicting in vivo performance of oral formulations administered to Parkinson’s patients [11]. The authors concluded that while there is a considerable amount of information regarding salivary secretion, gastric emptying, and oropharyngeal and esophageal passage, more knowledge is required in the area of motility patterns and pressures in the GI tract, as well as GI fluid composition of Parkinson’s patients. Such a knowledge gap makes it difficult to design a biorelevant dissolution method for antiparkinsonian drugs.

Taking into account the potential for new formulation development and the fact that ropinirole’s PK might be affected by both age- and disease-related physiological parameters, it is a good practice to have a PBPK model which can help explore a number of different clinical scenarios and understand whether the quality control dissolution data can be biopredictive for an in vivo dissolution. The purpose of this study was to develop a PBPK model for ropinirole prolonged-release formulation in order to test the hypothesis of biopredictiveness of dissolution data obtained via the USP method without consideration of changes in GI tract of Parkinson’s patients.

## 2. Materials and Methods

### 2.1. PBPK Model Development

All the modeling and simulation activities were conducted using Simcyp^®^ Simulator (V20, Certara, Sheffield, UK). Data for model development and verification was extracted from publicly available sources. Ropinirole mean concentration-time profiles were digitized using GetData Graph Digitizer [12], where necessary.

The PBPK model parameters are provided in Table 1 below.

### 2.2. Description of Distribution and Elimination

To reduce the potential interference of the absorption-related confounding factors and gain confidence in the ropinirole volume of distribution and systemic clearance, a PBPK model was initially developed for the immediate-release oral formulation [13]. The above-mentioned parameters, namely clearance and volume of distribution, were estimated using a bottom-up approach due to the fact that intravenous clinical data for this compound is scarce [25]. To describe ropinirole distribution, a full PBPK model was chosen based on the compound’s lipophilicity (logP = 2.7 [14]), the volume of distribution/bioavailability reported in the literature being around 7 L/kg, and the fact that the target for the drug action is located in a peripheral organ, namely CNS [26]. To incorporate clearance into the model, two approaches were considered: interspecies scaling and in vitro–in vivo extrapolation (IVIVE) [13]. The IVIVE approach has been chosen for the final model because systemic clearance predicted by the interspecies scaling did not properly describe multiple dosing scenarios. For illustration of the differences between two approaches, we simulated study No. 112771. It is worth noting that a fraction of the drug unbound in the incubation media (fu_inc_) was not specified in the in vitro study report and was fitted in Simcyp using Nelder–Mead minimization method, 0.3 (0.00; 1.00) as initial value (lower bound; upper bound, respectively), maximum number of iterations 100, and weighted least-squares objective function.

### 2.3. Description of the Absorption

After defining the volume of distribution and systemic clearance, the next step was to model ropinirole absorption from a 24-h prolonged-release formulation. For this purpose, the advanced dissolution, absorption, and metabolism (ADAM) model implemented in Simcyp Simulator was chosen. ADAM model consists of nine anatomically relevant compartments: stomach, seven compartments reflecting the small intestine, and the colon. The drug’s movement from one compartment to another is described as the first-order process. Additionally, the model allows accounting for intestinal efflux and metabolism. All the processes occurring in the GIT are described by the set of ordinary differential equations. The general form of the equations applicable for the small intestine is provided below [27]:

**Amount of solid mass available for dissolution (*A_S_*):**

(1) $$\frac{dA_{S,n}}{dt}=-\frac{dA_{diss,n}}{dt}-k_{t,n}A_{S,n}+k_{t,n-1}A_{S,n-1}+\frac{dA_{F,n}}{dt}\;$$

**The amount of drug dissolved (*A_D_*):**

(2) $$\frac{dA_{D,n}}{dt}=\frac{dA_{diss,n}}{dt}-\left(k_{deg,n}+ka_{n}+k_{t,n}\right)A_{D,n}+k_{t,n-1}A_{D,n-1}+\gamma_{n}CLu_{int-T,n}fu_{gut}C_{ent,n}$$

**The drug concentration in the enterocytes (*C_ent_*):**

(3) $$\frac{dC_{ent,n}}{dt}=\frac{1}{V_{ent,n}}\left(A_{diss,n}ka_{n}-C_{ent,n}Q_{ent,n}-fu_{gut}C_{ent,n}\left[CLu_{int-G,n}+CLu_{int-T,n}\right]\right)$$

where: *n* is the small intestine compartment, *n* = 1, 2, …, 7

$\frac{dA_{diss,n}}{dt}$ : Dissolution rate
$k_{t,n}$ : Transit time in the *n*-th segment of the small intestine
$A_{F,n}$ : Amount of solid mass trapped in the formulation and not available for dissolution immediately
$k_{deg,n}$ : Drug degradation rate constant (in lumen)
$ka_{n}$ : Absorption rate constant
$\gamma_{n}$ : The unit adjustment factor for the drug transported out of the enterocyte
$CLu_{int-T,n}$ : Efflux clearance from the enterocyte
$CLu_{int-G,n}$ : Metabolic clearance within the enterocyte
$fu_{gut}$ : Fraction of the drug unbound in the enterocyte
$C_{ent,n}$ : Drug concentration in the enterocyte
$V_{ent,n}$ : The volume of enterocytes in the segment
$Q_{ent,n}$ : Blood flow to the intestinal segment

In addition, ADAM model included Segregated Transit Time sub-model, which was used to define mean residence time for the fluid and dissolved drug in the stomach, small intestine, and colon.

### 2.4. Prolonged-Release Formulation

The prolonged-release formulation modeled in this study was Requip XL [9]. The tablet is based on Geomatrix technology [28] and consists of a slow-releasing active core sandwiched between two semi-permeable inactive layers. The list of typical excipients can be found in Requip XL Approval Package [9]. The complete release of the active substance from the formulation is achieved in 24 h.

### 2.5. Dissolution Data

All the dissolution-related processes were implemented into the model directly using quality control dissolution profiles extracted from GSK clinical study reports available at [29]. The details of the dissolution method were not disclosed in the published documents; however, it was stated that the dissolution profiles complied with USP <724> monograph requirements, allowing for the assumption of compendial conditions.

In addition, in a clinical pharmacology and biopharmaceutics review for Requip XL conducted by Center for Drug Evaluation and Research (hereinafter, CDER review) [9], the following dissolution method proposed by a Sponsor is described: USP Apparatus 2 with paddles, medium: citrate buffer at pH 4.0, paddle speed: 100 rpm. It is also stated that Requip XL dissolution was independent of the strength or the dissolution media (media with physiologically relevant pHs were tested), except for 0.1 M hydrochloric acid, where the drug release was slightly faster because of slightly impaired gelling of hypromellose in the acidic media.

It is important to highlight that the dissolution profiles, trial design, and concentration-time profiles for the model verification were coming from the same source. The dissolution profiles reported in the certificates of analysis included three timepoints which were used to fit Weibull function parameters *α* and *β*:

(4) $$F_{diss}=F_{max}\left(1-e^{-\frac{\left(t-lag\right)\beta }{\alpha }}\right)$$

assuming *F_max_* = 100% and lag time = 0. Alpha and beta parameters of the Weibull fit for each formulation are presented in Table 1.

**Figure 1:**
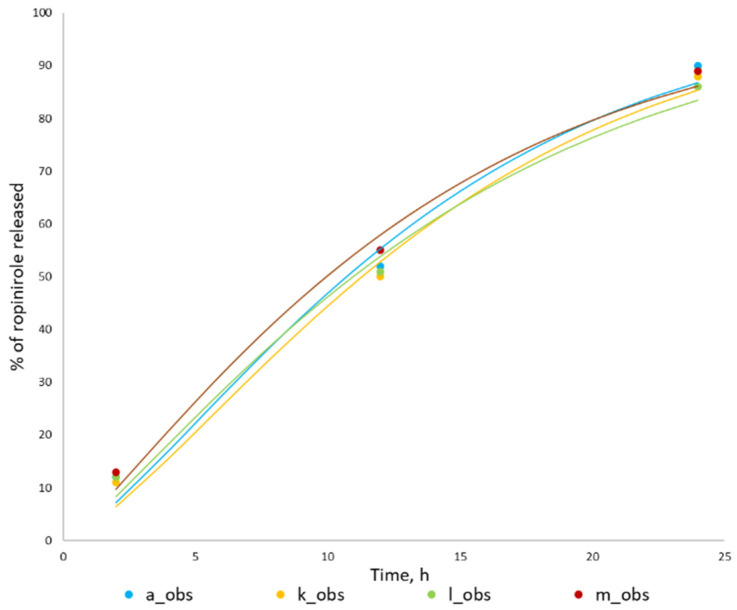
Typical dissolution profiles which were used in the PBPK model. Latin letters (a, k, l, m) indicate simulation ID (please see Table 2), while obs indicates extracted dissolution data and pred —Weibull fit.

The representative dissolution profiles together with the Weibull fits are provided in Figure 1 below.

Additionally, Study No. ROP109087 for 8 mg was simulated with the use of dissolution data extracted from CDER review: the profiles in citrate buffer of pH 4.0 and in phosphate buffer of pH 6.8, which included eight timepoints each. The results were compared with those obtained with the three-timepoint dissolution profile from the certificate of analysis.

### 2.6. Intestinal Permeability

It was assumed that the drug intestinal transport is passive, therefore the result of PAMPA study was used as the model input (apparent permeability (Papp) = 26.8 × 10^−6^ cm/s) [17]. The assumption is supported by a lack of reports providing information on ropinirole affinity to transporters [30]. Papp was scaled to the permeability in Caco-2 cell line based on the established correlation described in [30] and subsequently to human effective permeability (Peff) according to [31].

### 2.7. Fraction of Drug Unbound in the Enterocytes (fu_gut_)

Fraction of drug unbound, fu_gut_, was predicted by the Simcyp built-in model using the Rodgers and Rowland method (Method 2 on-screen in Simcyp Simulator) [32].

### 2.8. Intestinal Metabolism

Ropinirole is a soluble and permeable drug; therefore, intestinal metabolism is not of clinical relevance for this compound. Moreover, the main ropinirole metabolizing enzyme CYP1A2 is not expressed in the intestinal wall [23].

To summarize the model-building step, the assumptions of the ADAM model are provided below:

- The release from the formulation is the only rate-limiting factor in the ropinirole’s absorption.
- In vitro dissolution data, obtained by the quality control method, is biopredictive as for ropinirole’s behavior in the human GIT.
- There is no drug degradation in the intestinal lumen.
- The intestinal absorption is based on passive diffusion, and there are no efflux processes taking place at the intestinal wall.
- Ropinirole intestinal metabolism is negligible.
- The formulation provides stable active substance release in the Parkinson’s patients GIT environment.

### 2.9. Model Verification and Application

Clinical study results from the reports mentioned previously were used for model verification. During the simulation exercises, the trial design was followed exactly as in the clinical trials. Namely, number of patients, age ranges, and male-to-female ratios in virtual trials were consistent with those in clinical trials. Demographic, anatomical, and physiological parameters of virtual patients were generated by the software using Monte Carlo approach based on the population libraries available in the Simcyp. For simulation of trials in healthy volunteers, Sim-Healthy Volunteers library was used. This virtual population is based on real life data from subjects who participated in Phase I studies [33]. For the simulation of trials in Parkinson’s patients, Sim-NEurCaucasian population was used, considering the unavailability of the disease population. North European Caucasian population in Simcyp represents “general” rather than healthy population because it is difficult to define the latter, taking into account age-related changes in physiology together with minor and major health issues. The parameters of this population have been described previously [34]. The Geriatric population available in the simulator could not be used for simulation of the specified trials because the lower age limit of patients in the clinical trials was 34 years. The summary for trial design is provided in Table 2 below, including the indication of actual clinical trial populations versus virtual populations used in Simcyp.

It should be noted that healthy volunteers and patients in some cases administered ropinirole with domperidone. This combination does not bring any modification to the pharmacokinetics of either of them [35].

For the assessment of model performance, the simulated versus observed fold differences were calculated for Cmax, Tmax, and AUC (0–∞ or 0–24 depending on the study), along with the visual check of the concentration–time profiles. After the model verification step, we simulated the study ROP109087 in order to compare the drug exposure between the currently used group of patients (Sim-NEurCaucasian, aged 47–81) and three groups of geriatric population aged 65–75, 75–85, and 85–98 years old. For these simulation runs, we used Sim-Geriatric NEC population, which is based on Sim-NEurCaucasian population and takes into account age-dependent changes in physiological parameters [36].

## 3. Results

**Figure 2:**
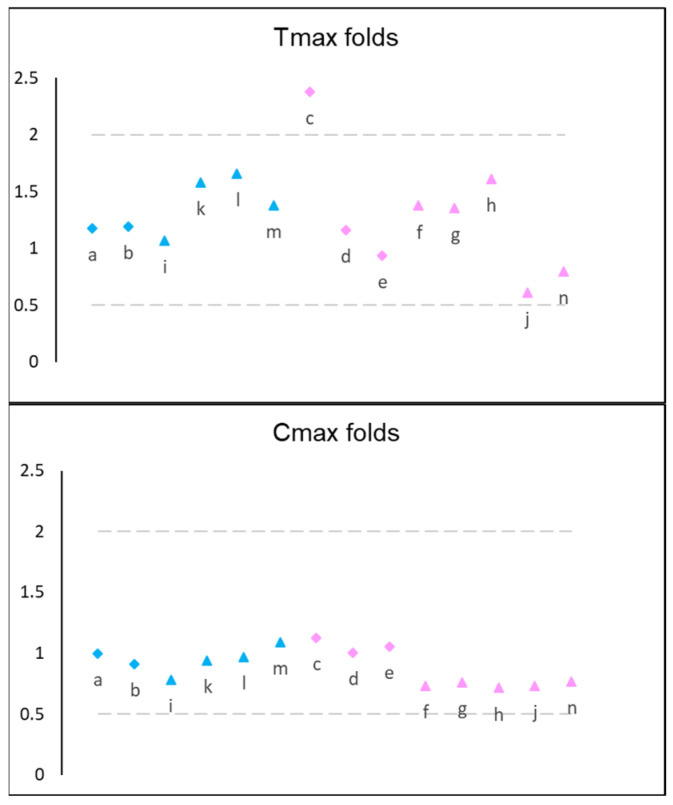

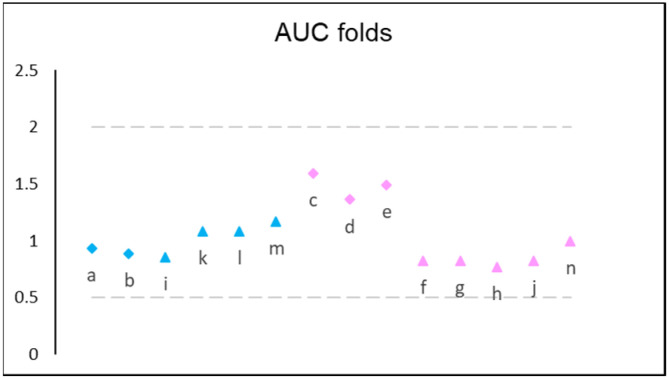
Plotted fold differences for simulated versus observed values. Diamonds indicate healthy volunteers, triangles—Parkinson’s patients, blue color—fasted state, pink color—fed state. Each symbol represents a fold value from each simulation included in Table 2. Latin letters indicate Simulation ID referenced in Table 2.

The model was verified by simulating five clinical trials and covered all the strengths available on the market (from 2 to 12 mg), as well as fasted and fed states. The simulated versus observed fold values for Tmax, Cmax, and AUC are provided graphically in Figure 2. All the values were within a two-fold range, which was the criteria for PBPK model performance assessment used in this study. The only exception was Tmax fold in study No. 101468-219 (1 mg), which was 2.37. For calculation of fold differences, mean, geometric mean, or median values were extracted from the studies (depending on availability in the report), and the respective values were generated by the simulator, both from the statistical perspective (for example, mean observed vs. mean simulated) and from the PK perspective (for example, AUC0-inf observed vs. AUC0-inf simulated).

**Figure 3:**
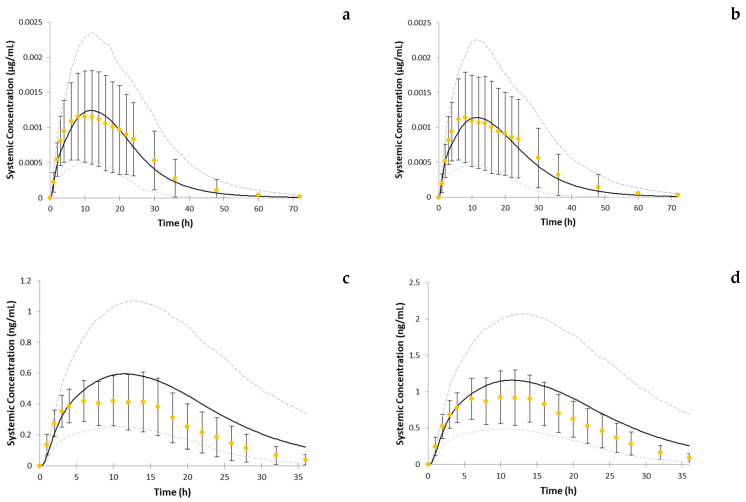

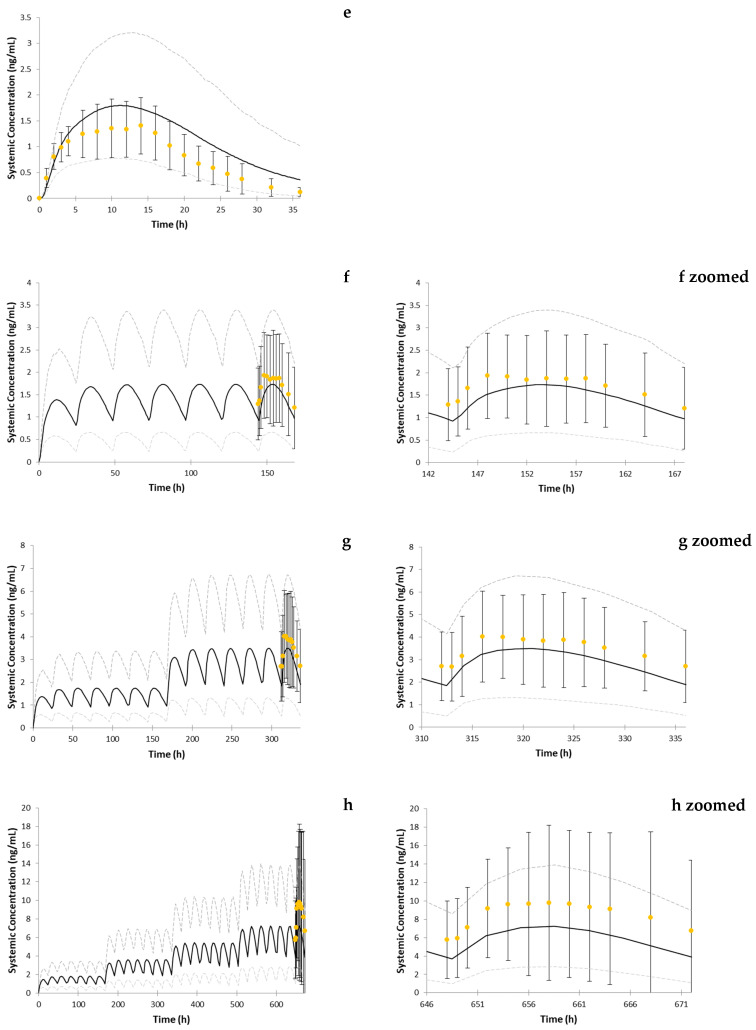

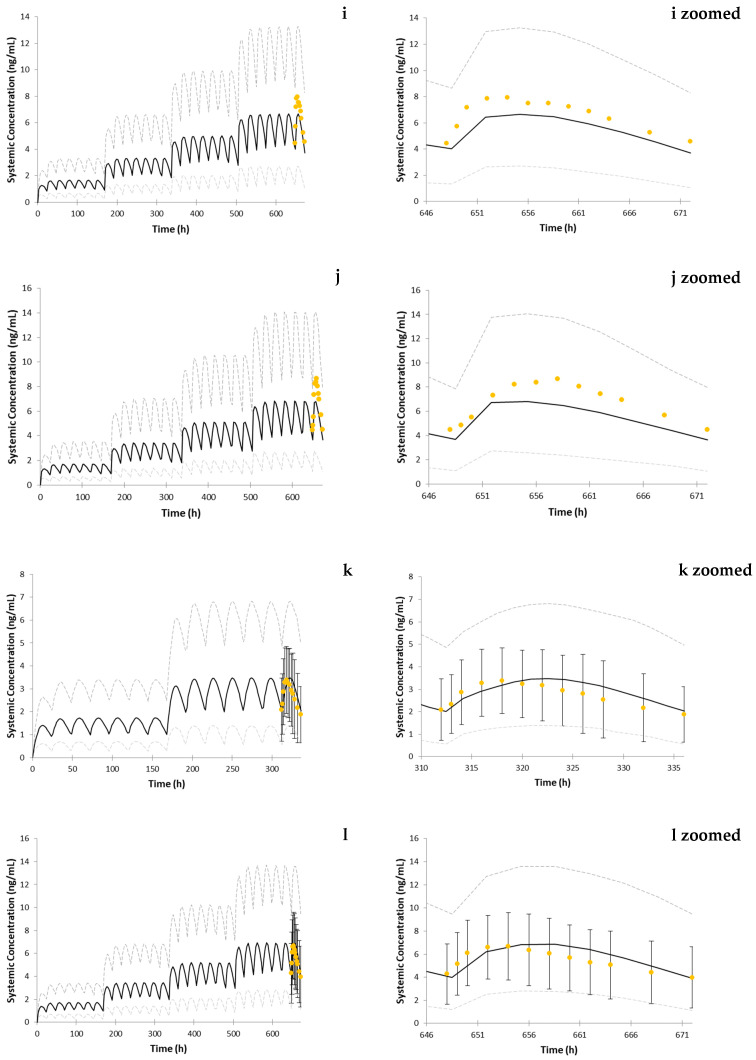

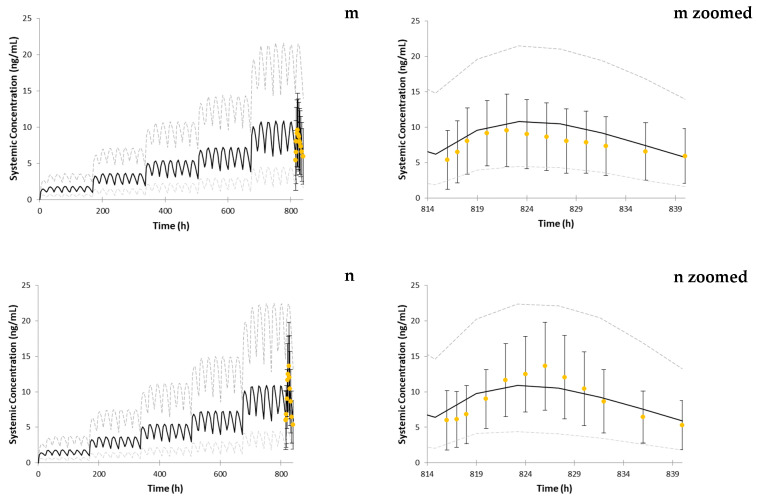
Mean observed (circles) and simulated (solid line) systemic plasma concentration–time profiles of ropinirole. Variability of the observed data is provided as SD if reported in the study; 5%th and 95%th percentiles are presented as grey dashed lines. Design of studies is provided in Table 2. Each Latin letter in this figure is a reference to the Simulation ID in Table 2. Multiple dose simulations additionally zoomed (e.g., f–n zoomed) in the time period where the observed data was available.

**Figure 4:**
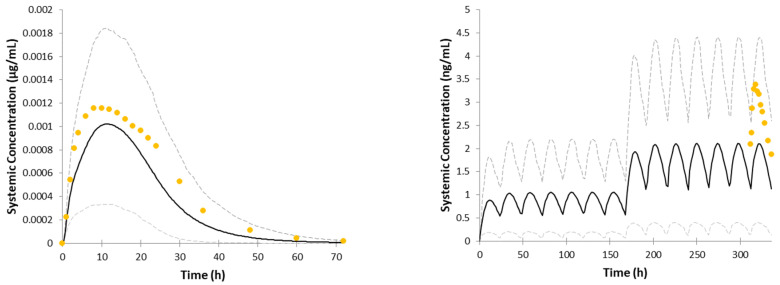
Mean observed (circles) and simulated (solid line) systemic plasma concentration–time profiles of ropinirole (5%th and 95%th percentiles are presented as grey dashed lines). Simulations of GSK study No. 112771 (2 mg, Aranda site) and study No. ROP109087 (4 mg) with the use of in vivo clearance obtained by interspecies scaling.

**Figure 5:**
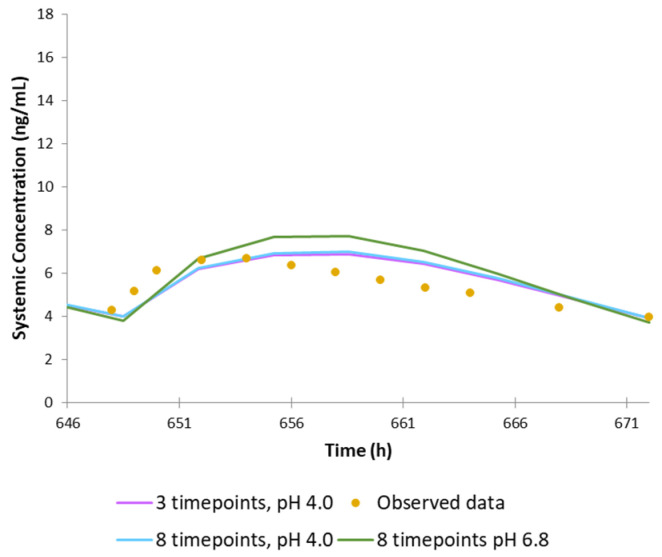
Mean observed (circles) and simulated (solid line) systemic plasma concentration–time profiles of ropinirole (5%th and 95%th percentiles are presented as grey dashed lines). Comparison of the simulations of GSK study No. ROP109087 (8 mg) with the use of three-timepoint dissolution profile, eight-timepoint dissolution profile obtained at pH 4.0, and eight-timepoint dissolution profile obtained at pH 6.8.

**Figure 6:**
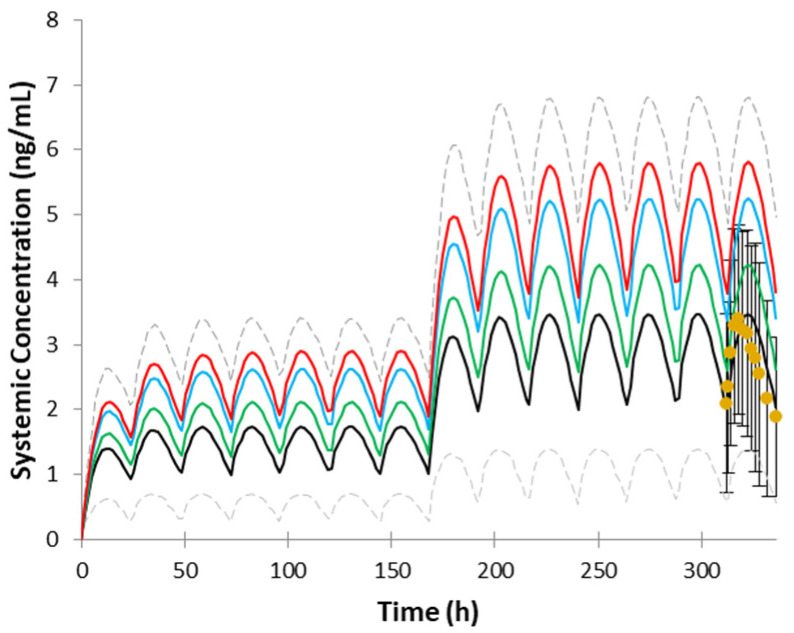
Mean observed (circles) and simulated (solid line) systemic plasma concentration–time profiles of ropinirole (5%th and 95%th percentiles are presented as grey dashed lines). Simulation of study No. ROP109087 (4 mg) using Sim-NEurCaucasian population, 47–81 years old (black line); Sim-Geriatric population 65–75 years old (green line); Sim-Geriatric population 75–85 years old (blue line); and Sim-Geriatric population 85–98 years old (red line). Dotted lines represent 5th and 95th percentiles, circles represent observed data. Data presented as mean and SD.

The visual checks are provided in Figure 3. Figure 3a–e represents single-dose studies whereas Figure 3f–n represents multiple-dose studies. The multiple-dose simulations are additionally zoomed in the timeframe where the observed data were available. Figure 4 illustrates the model performance when IV clearance was predicted based on interspecies scaling and used in the model as a whole value. The simulation results with the use of different dissolution profiles are provided in Figure 5. Results of trial simulations in geriatric population are presented in Figure 6.

## 4. Discussion

Physiologically based pharmacokinetic and absorption modeling are being used by industry and regulatory bodies to address various scientifically challenging questions. The PBPK models describing mechanistic absorption process of orally taken drugs have been less commonly reported as part of regulatory approvals in comparison to other application areas. According to Zhang et al., the distribution of PBPK submissions by application areas in 2018–2019 included predominantly the DDI-related PBPK analyses (56%) of the total number of applications, while absorption accounted for 7% [37]. For the current project, detailed, case-by case-analysis was applied, yet there is high confidence in the predictions for the BCS class I drugs [38,39]. The purpose of this study was to develop and verify a PBPK model for the BCS class 1 compound ropinirole, where the model is supposed to be able to predict ropinirole exposure of prolonged-release formulation for oral administration in various doses to the patient population.

The key PK parameters of the model were already verified in the number of simulations for the immediate-release formulation described recently in [13]. As ropinirole is mostly eliminated by enzymatic metabolism in the liver, special attention was given to the IVIVE method of the clearance prediction after the interspecies scaling approach occurred to not be sufficient for the prediction of most of the multiple-dose studies. Please refer to the predictions obtained with the use of in vivo clearance from interspecies scaling after IV administration in Figure 4. For the comparison with IVIVE approach, please see Figure 3a,k.

The simulations of the same studies with the use of the IVIVE approach are presented in Figure 3a (single-dose study) and Figure 3k (multiple-dose study). We hypothesized that the main reason that the IVIVE approach was more efficient lies in accounting for the inter-individual variability in the clearance-related physiological parameters. For this reason, for clearance prediction in the final PBPK model, an IVIVE approach was used.

The absorption of ropinirole from modified release formulation was modeled using a less mechanistic approach: the dissolution was inputted into the model directly using quality control data and effective human permeability was predicted using an empirical correlation equation. Dissolution data are the key input parameter for the PBPK model, and in cases where the dissolution data are used in the model “as is” rather than modeled mechanistically, the assumption about their relevance to the in vivo dissolution has to be made. For reaching the point where this assumption is confirmed/rejected, a strong confidence in distribution and elimination parameters is needed. Due to the lack of intravenous data for ropinirole, this aim was achieved by conducting the modeling and simulation (M&S) activities for the immediate-release formulation, where absorption was not a rate-limiting step. Only after successful simulation of number of single-dose and multiple-dose clinical studies in healthy volunteers and patient populations could we fix the distribution and elimination parameters and move to the following step: M&S for the prolonged-release formulation, where the release from the formulation was a rate-limiting step. The model was initially developed for healthy volunteers and was subsequently extrapolated to the Parkinson’s patients. In all cases, quality control dissolution profiles were used as an input to the absorption model. While looking at simulation results for healthy volunteers and Parkinson’s patients, it can be observed that the performance of the model is similar both in Parkinson’s patients and healthy volunteers. Please see Figure 3a–e for the simulation of studies with healthy volunteers and Figure 3f–n for the simulation of studies in Parkinson’s patients. The fact that the model, which was developed for healthy volunteers, could be extrapolated to the patient population without any modification of physiological parameters besides those that are age-related allows for the following conclusions: (1) the quality control dissolution data are biopredictive for the in vivo situation; (2) even if there are changes in the gastro-intestinal parameters of Parkinson’s patients, these changes do not influence the release of the drug from the prolonged-release formulation, which is based on Geomatrix technology. We assumed no impact of fluid dynamics, pressures, or motility patterns in patients’ GIT on release patterns of the formulation. This still allowed the model predictions to align with the PK profiles observed in patients, thus indirectly confirming that these parameters would not have a clinically significant effect on formulation behavior in vivo. In other words, it can be concluded that the formulation is stable in the Parkinson’s patient GI environment.

Because the dissolution profiles used in the simulations composed of three timepoints only (2, 12, and 24 h), it was decided to conduct extra simulations using the dissolution data from CDER review. We compared three simulation scenarios: (1) with the use of a three-timepoint profile from the certificate of analysis (it was assumed that the profile was obtained at pH 4.0); (2) with the use of eight-timepoint profile from CDER review obtained at pH 4.0; (3) with the use of eight-timepoint profile from CDER review obtained at pH 6.8. The results of these simulations, which are provided in Figure 5, suggest that the three-timepoint profile is reflective of the profile with eight-timepoints. In addition, despite it being claimed that dissolution of the formulation is pH-independent, our simulation run on dissolution data obtained at different pH shows that the model is sensitive to these changes and that the profile obtained at pH 4.0 gives a more realistic prediction than the profile obtained at pH 6.8.

Parkinson’s disease is rarely diagnosed at age under 40 and its incidence increases with age [1]. Older patients are generally underrepresented in the clinical trials; therefore, PBPK can be a useful alternative approach for predicting drug pharmacokinetics [37]. The simulation of trials in the three groups of geriatric population have indicated that drug exposure increases with age, which is expected based on the fact that ropinirole is mainly metabolized in the liver and metabolic rates are slower in older patients [26]. The increased drug exposure in older patients has to be taken into account during pharmacokinetic optimization of ropinirole’s therapy [4].

## 5. Conclusions

In this study, ropinirole PBPK model for the prolonged-release formulation intended for exploring the potential clinical scenarios while developing a new formulation was built and verified. In vitro data obtained by the quality control method implemented into the ADAM model adequately described the in vivo behavior of the formulation both in healthy volunteers and in Parkinson’s patients. The assumption about the stable release from the formulation in the GIT of the patient population was indirectly confirmed. It is worth noting that this PBPK model is formulation specific; however, we believe that it can be used for our research purposes after the mechanical stability (e.g., resistance to pressure) of the new formulation is established.

The performed simulations allowed us to additionally verify in vitro kinetic parameters for ropinirole metabolism, as well as its metabolic pathways. In this study, most of the datasets were multiple-dose studies, and new strengths, which are not available in the immediate-release case, were used.

## Figures and Tables

**Table 1:** Ropinirole PBPK model parameters used for the prolonged-release formulation [13].

Parameter	Description	Value	Reference
**Ropinirole physicochemical properties and blood binding**			
MW (g/mol)	Molecular weight	260.38	Chemicalize.com
LogP_o:w_	Neutral species octanol: buffer partition coefficient	2.7	[14]
Type of the compound		Monoprotic base	
pKa	Dissociation constant	9.79	[15]
B/P	Blood-to-plasma partition ratio	1.09	[16]
fu	Fraction unbound in plasma	0.68	[16]
**Absorption model**	Advanced Dissolution, Absorption, and Metabolism (ADAM) model		
fa	Fraction available from a dosage form	0.99	Simcyp^®^ predicted
ka (h^−1^)	First-order absorption rate constant	2.19	Simcyp^®^ predicted
P_app_ (PAMPA, 10^−6^ cm/s)	Apparent permeability in PAMPA	26.8	[17]
P_eff_, man (10^−4^ cm/s)	Effective human jejunum permeability	5.01	Simcyp^®^ predicted
Weibull fit parameters	alpha	33.70	Study 112771, Aranda site [18]
	beta	1.33	
	alpha	28.95	Study 112771, Crawley site [18]
	beta	1.23	
	alpha	33.66	Study 101468/219, 1 mg [19]
	beta	1.34	
	alpha	30.86	Study 101468/219, 2 mg [19]
	beta	1.29	
	alpha	29.57	Study 101468/219, 3 mg [19]
	beta	1.20	
	alpha	33.71	Study 101468/165, 2 mg [20]
	beta	1.35	
	alpha	24.12	Study 101468/165, 4 mg [20]
	beta	1.22	
	alpha	34.02	Study 101468/165, 8 mg [20]
	beta	1.37	
	alpha	17.14	Study101468/164 [21]
	beta	1.08	
	alpha	38.44	Study ROP109087, 4 mg [22]
	beta	1.35	
	alpha	26.55	Study ROP109087, 8 mg [22]
	beta	1.22	
	alpha	22.24	Study ROP109087, 12 mg [22]
	beta	1.19	
**Distribution Model**	Full PBPK		
V_ss_ (L/kg)	Volume of distribution at steady state	3.37	Simcyp^®^ predicted Method 2
Elimination			
Enzyme kinetic parameters for IVIVE			
*N-despropylation*	Enzyme	Value	
V_max_ (nmol/h/mg)	CYP1A2	7.83	[23]
K_m_ (µmol)	CYP1A2	34.63	
V_max_ (nmol/h/mg)	CYP3A4	523.33	
K_m_ (µmol)	CYP3A4	2700.00	
*Hydroxylation*			
V_max_ (nmol/h/mg)	CYP1A2	6.93	
K_m_ (µmol)	CYP1A2	45.87	
V_max_ (nmol/h/mg)	CYP3A4	255.33	
K_m_ (µmol)	CYP3A4	3933.33	
fu_mic_	Fraction unbound in an in vitro microsomal preparation	0.39	Estimated based on dataset from Study No. 101468/197 [24]

**Table 2:** Summary of the clinical trial design used in the simulations.

Study ID * and Reference	Simulation ID	Clinical Study Population	Virtual Population	Subject Age	n of Subjects	PK Assessment Dose, mg	Dosing Regimen	Prandial State
112771 [18]	a (Aranda site) b (Crawley site)	HV	Sim-Healthy Volunteers	18–50	50	2	QD	Fasted
101468/219 [19]	c	HV	Sim-Healthy Volunteers	18–44	31–33	1 2 3	QD QD QD	Fed
	d							
	e							
101468/165 [20]	f	PARKD	Sim-NEurCaucasian	47–87	25	2	QD × 7 days **	
	g					4	2 mg QD × 7 days, 4 mg QD × 7 days **	
	h					8	2 mg QD × 7 days 4 mg QD × 7 days 6 mg QD × 7 days 8 mg QD × 7 days **	
101468/164 [21]	i	PARKD	Sim-NEurCaucasian	34–80	21	8	2 mg QD × 7 days 4 mg QD × 7 days 6 mg QD × 7 days 8 mg QD × 7 days **	Fasted
	j							Fed
ROP109087 [22]	k	PARKD	Sim-NEurCaucasian	47–81	27	4	2 mg QD × 7 days 4 mg QD × 7 days **	Fasted
	l					8	2 mg QD × 7 days 4 mg QD × 7 days 6 mg QD × 7 days 8 mg QD × 7 days **	
	m					12	2 mg QD × 7 days, 4 mg QD × 7 days 6 mg QD × 7 days 8 mg QD × 7 days 12 mg QD × 7 days **	Fasted
	n							Fed

* According to the GSK Clinical Trials Register; ** PK assessment on the last day of the dosing period or at steady state for the highest dose.

## References

[1] Hayes M.T. (2019). Parkinson’s Disease and Parkinsonism. Am. J. Med..

[2] Samii A., Nutt J.G., Ransom B.R. (2004). Parkinson’s disease. Lancet.

[3] Homayoun H. (2018). Parkinson Disease. Ann. Intern. Med..

[4] Contin M., Riva R., Albani F., Baruzzi A. (1996). Pharmacokinetic optimisation in the treatment of Parkinson’s disease. Clin. Pharm..

[5] Nyholm D. (2006). Pharmacokinetic optimisation in the treatment of Parkinson’s disease: An update. Clin. Pharm..

[6] Dobson A.M., Cuellar N. (2006). Use of ropinirole in RLS. Nurse. Pract..

[7] Latt M.D., Lewis S., Zekry O., Fung V.S.C. (2019). Factors to Consider in the Selection of Dopamine Agonists for Older Persons with Parkinson’s Disease. Drugs Aging.

[8] Jenner P. (2004). Avoidance of dyskinesia: Preclinical evidence for continuous dopaminergic stimulation. Neurology.

[9] Drug Approval Package: Requip XL (Ropinirole) NDA #022008. https://www.accessdata.fda.gov/drugsatfda_docs/nda/2008/022008_requip_toc.cfm.

[10] Stillhart C., Vučićević K., Augustijns P., Basit A.W., Batchelor H., Flanagan T.R., Gesquiere I., Greupink R., Keszthelyi D., Koskinen M. (2020). Impact of gastrointestinal physiology on drug absorption in special populations—An UNGAP review. Eur. J. Pharm. Sci..

[11] Wollmer E., Klein S. (2017). A review of patient-specific gastrointestinal parameters as a platform for developing in vitro models for predicting the in vivo performance of oral dosage forms in patients with Parkinson’s disease. Int. J. Pharm..

[12] http://getdata-graph-digitizer.com.

[13] Shuklinova O. Development of Physiologically Based Pharmacokinetic Model for the Immediate Release Ropinirole Tablets—Acta Poloniae Pharmaceutica—Drug Research—Czasopisma Naukowe. https://ptfarm.pl/wydawnictwa/czasopisma/acta-poloniae-pharmaceutica/110/-/29108.

[14] Adlard M., Okafo G., Meenan E., Camilleri P. (1995). Rapid estimation of octanol–water partition coefficients using deoxycholate micelles in capillary electrophoresis. J. Chem. Soc. Chem. Commun..

[15] Coufal P., Stulík K., Claessens H.A., Hardy M.J., Webb M. (1998). Determination of the dissociation constants of ropinirole and some impurities and their quantification using capillary zone electrophoresis. J. Chromatogr. B Biomed Sci. Appl..

[16] Swagzdis J.E., Wittendorf R.W., DeMarinis R.M., Mico B.A. (1986). Pharmacokinetics of dopamine-2 agonists in rats and dogs. J. Pharm. Sci..

[17] Iwasaki S., Yamamoto S., Sano N., Tohyama K., Kosugi Y., Furuta A., Hamada T., Igari T., Fujioka Y., Hirabayashi H. (2019). Direct Drug Delivery of Low-Permeable Compounds to the Central Nervous System Via Intranasal Administration in Rats and Monkeys. Pharm. Res..

[18] GSK—A Study Conducted in Healthy Subjects to Demonstrate Bioequivalence between Ropinirole Prolonged Release Tablets Manufactured at Crawley and Aranda. https://www.gsk-studyregister.com/en/trial-details/?id=112771.

[19] GSK—An Open Label, Randomised, Five-Way Crossover Single-Dose Pharmacokinetic Study to Assess Dosage Strength Equivalence of Ropinirole CR in Healthy Male and Female Volunteers. https://www.gsk-studyregister.com/en/trial-details/?id=101468/219.

[20] GSK—An Open-Label, Up-Titration Study to Assess the Dose Proportionality of Ropinirole Controlled Release (CR) and to Demonstrate the Bioequivalence of Ropinirole CR (1 × 8 mg) Compared to the Ropinirole CR (4 × 2 mg) in Parkinson’s Disease Patients Not Receiving Other Dopaminergic Therapies. https://www.gsk-studyregister.com/en/trial-details/?id=101468/165.

[21] GSK—An Open Study to Investigate the Effect of Food on the Pharmacokinetics of Ropinirole from a CR Formulation in Healthy Male Volunteers. [Type ??]. https://www.gsk-studyregister.com/en/trial-details/?id=101468/164.

[22] GSK—Parkinson’s Disease Patient Study on Absorption, Distribution, Metabolism and Excretion of Ropinirole. https://www.gsk-studyregister.com/en/trial-details/?id=ROP109087.

[23] Bloomer J.C., Clarke S.E., Chenery R.J. (1997). In vitro identification of the P450 enzymes responsible for the metabolism of ropinirole. Drug Metab. Dispos..

[24] GSK—An Open Study to Compare the PK and Tolerability of Ropinirole Administered as 5 Different New Formulations with the Standard, Marketed Formulation in Healthy Volunteers. https://www.gsk-studyregister.com/en/trial-details/?id=101468/197.

[25] GSK—An Open Study to Investigate the Tolerance and Preliminary Pharmacokinetics of Single Intravenous Doses of 100, 200, 400, 600 and 800 mg SK&F 101468 Following Domperidone Pre-Treatment (20 mg t.i.d.) in Healthy Male Volunteers. https://www.gsk-studyregister.com/en/trial-details/?id=101468/009.

[26] Kaye C.M., Nicholls B. (2000). Clinical pharmacokinetics of ropinirole. Clin. Pharm..

[27] Jamei M., Turner D., Yang J., Neuhoff S., Polak S., Rostami-Hodjegan A., Tucker G. (2009). Population-based mechanistic prediction of oral drug absorption. AAPS J..

[28] Conte U., Maggi L. (1996). Modulation of the dissolution profiles from Geomatrix multi-layer matrix tablets containing drugs of different solubility. Biomaterials.

[29] https://www.gsk-studyregister.com.

[30] Fujikawa M., Ano R., Nakao K., Shimizu R., Akamatsu M. (2005). Relationships between structure and high-throughput screening permeability of diverse drugs with artificial membranes: Application to prediction of Caco-2 cell permeability. Bioorg. Med. Chem..

[31] Sun D., Lennernas H., Welage L.S., Barnett J.L., Landowski C.P., Foster D., Fleisher D., Lee K.-D., Amidon G.L. (2002). Comparison of human duodenum and Caco-2 gene expression profiles for 12,000 gene sequences tags and correlation with permeability of 26 drugs. Pharm. Res..

[32] Rodgers T., Leahy D., Rowland M. (2005). Physiologically based pharmacokinetic modeling 1: Predicting the tissue distribution of moderate-to-strong bases. J. Pharm. Sci..

[33] Jamei M., Marciniak S., Feng K., Barnett A., Tucker G., Rostami-Hodjegan A. (2009). The Simcyp population-based ADME simulator. Expert. Opin. Drug Metab. Toxicol..

[34] Howgate E.M., Rowland Yeo K., Proctor N.J., Tucker G.T., Rostami-Hodjegan A. (2006). Prediction of in vivo drug clearance from in vitro data. I: Impact of inter-individual variability. Xenobiotica.

[35] de Mey C., Enterling D., Meineke I., Yeulet S. (1991). Interactions between domperidone and ropinirole, a novel dopamine D2-receptor agonist. Br. J. Clin. Pharmacol..

[36] Chetty M., Johnson T.N., Polak S., Salem F., Doki K., Rostami-Hodjegan A. (2018). Physiologically based pharmacokinetic modelling to guide drug delivery in older people. Adv. Drug Deliv. Rev..

[37] Zhang X., Yang Y., Grimstein M., Fan J., Grillo J.A., Huang S.-M., Zhu H., Wang Y. (2020). Application of PBPK Modeling and Simulation for Regulatory Decision Making and Its Impact on US Prescribing Information: An Update on the 2018-2019 Submissions to the US FDA’s Office of Clinical Pharmacology. J. Clin. Pharmacol..

[38] Wagner C., Zhao P., Pan Y., Hsu V., Grillo J., Huang S.M., Sinha V. (2015). Application of Physiologically Based Pharmacokinetic (PBPK) Modeling to Support Dose Selection: Report of an FDA Public Workshop on PBPK. CPT Pharmacomet. Syst. Pharm..

[39] Wu F., Shah H., Li M., Duan P., Zhao P., Suarez S., Raines K., Zhao Y., Wang M., Lin H. (2021). Biopharmaceutics Applications of Physiologically Based Pharmacokinetic Absorption Modeling and Simulation in Regulatory Submissions to the U.S. Food and Drug Administration for New Drugs. AAPS J..

